# Current Strategies in the Prevention and Management of Infection in Open Fractures

**DOI:** 10.26502/josm.511500198

**Published:** 2025-05-05

**Authors:** Laura Roberts, Mohamed Radwan Ahmed, Devendra K. Agrawal

**Affiliations:** Department of Translational Research, College of Osteopathic Medicine of the Pacific, Western University of Health Sciences, Pomona, California 91766, USA

**Keywords:** Bacteriophage, Bioelectric dressings, Biofilm, Debridement, Delayed healing, External fixation, Infection, Internal fixation, Local antibiotics, Open fracture

## Abstract

Open fractures are complex injuries that significantly increase the risk of infection and complications such as delayed healing, nonunion, and chronic osteomyelitis. Infections rates remain high, particularly in severe cases involving extensive soft tissue damage and contamination. This is due to a variety of factors involving the patient, environment and bacteria. This article critically reviewed the classification, common pathogens, and complications associated with open fractures, emphasizing the challenges posed by biofilms, antibiotic resistance, and host factors such as diabetes and immunosuppression. Current management strategies, including early antibiotic administration, surgical debridement, and wound care, are examined alongside emerging therapies such as continuous local antibiotic perfusion, antibiotic-coated implants, bacteriophage therapy, and bioelectric dressings. These approaches show promise in reducing infection rates, enhancing patient outcomes, and addressing the limitations of traditional treatments. However, there are gaps in understanding their long-term efficacy, especially in high-risk populations. Future research should focus on personalized protocols, combination therapies, and clinical trials to reduce the burden of infection-related complications in open fracture management.

## Introduction

Open fractures are severe orthopedic injuries where bone pierces through the skin, thereby exposing the bone and soft tissues to external contaminants. This exposure significantly increases the risk of bacterial infection, which can lead to complications such as delaying healing, chronic osteomyelitis, cellulitis, sepsis, prolonged hospitalization and, in severe cases, amputation [1,2]. The consequences of such infections extend beyond the physical implications, creating significant emotional distress for patients and placing a heavy financial burden on both individuals and healthcare systems. Despite advances in surgical techniques and infection prevention strategies, infection rates in open fractures remain persistently high. This issue is worsened in high-grade contaminated fractures stemming from traumatic events, such as road accidents or agricultural injuries [3].

The greater the damage to bone and soft tissues, the higher the risk of the infection. This is due to compromised blood supply, reducing oxygen and nutrient delivery to the affected area, as well as increased exposure to contaminants to the external environment. Severe soft tissue loss or bone defects can lead to prolonged healing times, with increases the time for infection to develop. Greater tissue destruction can also lead to areas of necrosis in which bacteria can thrive. Weakened bone fragments act as breeding ground for infection, which also makes it difficult for antibiotics and immune cells to reach affected areas [4–6].

Each year, there are approximately 6 million fractures in the U.S., with nearly 4% classified as open fractures (around 240,000 cases) [7]. The standard of care for open fractures involves early administration of intravenous antibiotics, surgical debridement, and wound irrigation, followed by fracture stabilization. However, despite these interventions, fracture-related infections (FRIs) continue to represent a significant challenge in the management of open fractures [7]. Biofilms can develop on implants and create a barrier that can impact the effectiveness of antibiotics. Additionally, the emergence of multi-drug-resistant bacteria poses further difficulties, hindering the healing process and prolonging the time required for recovery [7,8].

Given the high morbidity and socioeconomic burden associated with infection in open fractures, considerable research has focused on optimizing treatment protocols to reduce infection rates and improve patient outcomes. The following review aims to evaluate current prophylactic strategies, treatment protocols, and emerging therapies in the management of open fracture-related infections.

## Classification of Open Fractures

**Figure 1:**
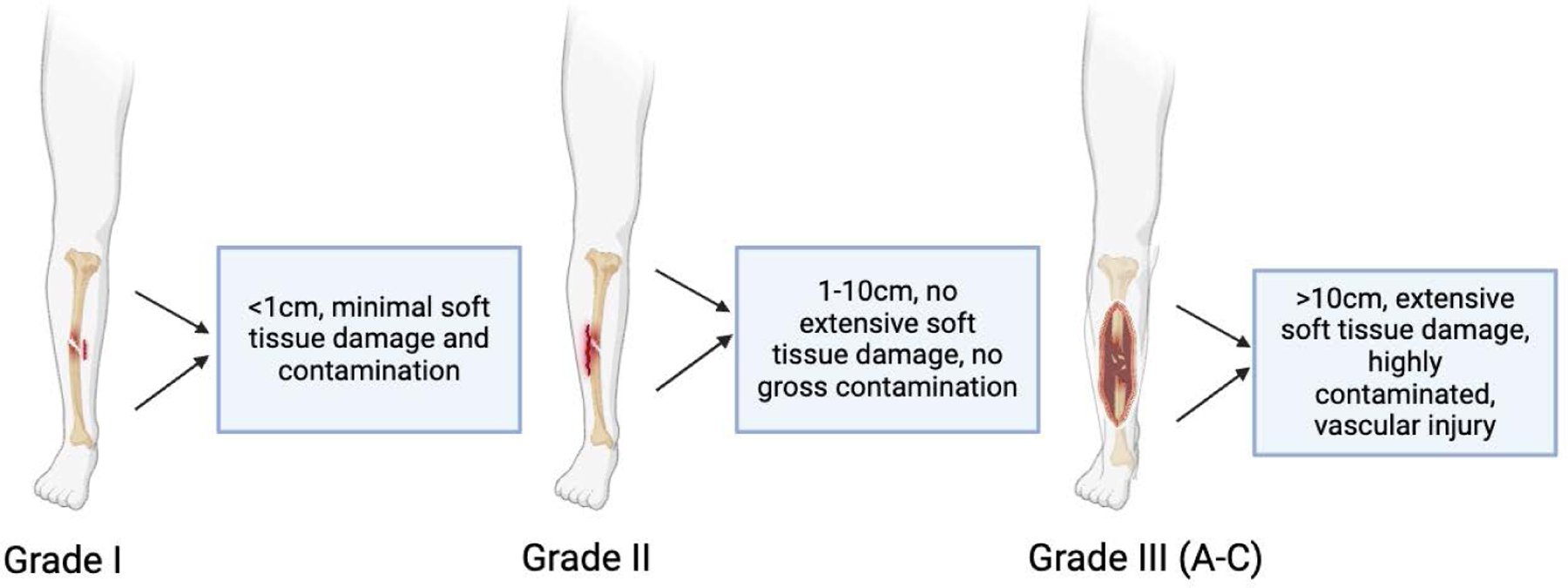
The schematic diagram showing system grades of open fractures by severity, as shown by the size, tissue damage and contamination, from Type I to Type II to Type III. Please note that Type III open fractures are further divided into A, B, and C, depending on the soft tissue coverage, soft tissue injury, and arterial injury.

While there is debate in some aspects of the treatment of open fractures, there is little debate over the classification of them. Open fractures are classified based on Gustilo-Anderson Classification (GAC) system, which categorizes based on size of the wound, degree of soft tissue injury, contamination, and presence or absence of vascular compromise [9]. This widely used system grades open fractures by severity from Type I to Type II to Type III (Figure 1). Type III fractures have a much higher risk of infection and poorer prognosis [9]. Type I fractures are classified with a wound less than 1 cm in length, low energy, and with minimal contamination and soft tissue damage. The infection risk is relatively low [9]. Type II open fractures contain a wound greater than 1 cm but less than 10 cm in length, without extensive soft tissue damage or flaps or without gross contamination. However, there is an infection risk associated with Type II [9]. Type III open fractures are split up into type III A, B, or C. They have extensive soft-tissue damage, high contamination, and can be associated with vascular injury (Figure 1). Type IIIA has adequate soft tissue coverage despite usually being over 10 cm [9,10]. Type IIIB is associated with extensive soft tissue injury with periosteal stripping and bone exposure. These require flap coverage. Finally, type IIIC is associated with an arterial injury that requires repair [9,10]. Classifying fractures in this way allows it to be used as a guide to antibiotic therapy as well as a predictor of outcomes such as delayed healing and non-unions. Proper classification is important, as higher-grade fractures, particularly Type III, are associated with significantly increased infection risks and poorer prognoses compared to lower grade fractures [9,10].

One drawback of this method is classifying an injury before the initial surgical debridement and potentially leading to an underestimate of the extent of the injury. However, according to a large multicenter randomized study, the initial misclassification of type III fractures as a type II did not significantly increase the risk of surgical site infections. Despite the different study results, correct classification can impact antibiotic therapy, surgical planning, and predict a patient’s prognosis [11].

## Common Organisms Associated with Open Fractures

Identifying the most common pathogens involved in fracture-related infections is helpful for guiding effective prevention strategies and optimizing treatment protocols. The most common pathogens that are detected in open fractures include Staphylococcus aureus and gram-negative bacteria [12,13]. One study found the most common pathogen with fracture related infections of the tibia or femur to be Staphylococcus aureus and then staphylococcus epidermidis. Non-epidermidis coagulase-negative staphylococci was also isolated along with polymicrobial infections with gram negative bacilli being most common. Finally, they isolated methicillin-resistant S. aureus (MRSA) [12]. A multicenter prospective study examined patients with high-energy open tibial fractures and found that a significant portion had positive cultures at the time of closure or coverage. Nearly half of the cultures contained gram-negative rods, with Enterobacter being the most frequently identified, followed by Pseudomonas [14]. Another study investigated patients with deep surgical site infections following fractures and found that most monomicrobial infections involved MRSA, methicillin-sensitive Staphylococcus aureus, and coagulase-negative Staphylococcus. In polymicrobial infections, gram-negative rods, including Enterobacter and Pseudomonas, were also commonly detected [15]. Many of the same types of infections are seen in open fractures. By gaining insight into the types of infections seen most in open fractures, preventative measures and treatment strategies could be tailored according to the characteristics of fractures. Further multicenter prospective studies could provide more comprehensive insights into resistance patterns and guide more precise therapeutic measures.

## Non-Unions and Delayed Healing

Common complications following open fractures and infections consist of non-union and delayed healing. The reasons for both are multifactorial. Non-union is when a fracture does not heal as expected, usually due to infection or inadequate blood supply. It can then require prolonged treatments or repeat surgeries [16]. Delayed healing can also require additional surgeries and complicate recovery. Infection, inadequate fracture stabilization, and compromised immune function can lead to delay healing [17]. One study found that over four decades (years 1977 to 2017) the rate of nonunion following open tibia fractures ranged from 13 to 17% [17]. Despite advances in care, the range has stayed consistent over 40 years. Another study found a similar nonunion rate of 17% with delayed healing in this study found in 8% of fractures [18]. With consistent rates, nonunion and delayed healing following open fractures can have significant consequences for patients, such as leading to prolonged disability or chronic pain. They may need more surgical intervention, therefore delaying recovery time and increasing healthcare costs. Addressing the underlying causes of non-union and delayed healing remains an important focus in fracture management.

## Management of Open Fractures

There are a variety of ways to treat open fractures. The management of infections following open fractures involves a multifaceted approach to prevent infection, promote fracture healing, and restore function. Some of the key components include early administration of antibiotics, surgical debridement and irrigation, wound management, local antibiotic delivery, and fracture stabilization. The open fracture should be cleaned from any contamination or foreign objects and, if possible, the reduction maneuver should reposition the bone beneath the soft tissue envelope. Loose bone fragments that are contaminated or lack blood supply should not be deliberately placed back into the open fracture wound. Instead, they should be wrapped in a sterile saline-soaked dressing and sent with the patient, allowing the surgeon to assess their importance for understanding and treating the fracture pattern. Then, to minimize motion, the fracture should be splinted, even in the deformed position if the reduction is unsuccessful [1].

Stabilizing the fracture with surgical fixation can help promote healing and reduce the risk of infection. It can help minimize further soft tissue trauma by reducing movement at the fracture site, enhance vascular supply through a stabilized blood flow, proper alignments help to reduce dead space, allow early wound management, enabling early mobilization, and supporting antibiotic penetration [19–21].

There is debate over plate fixation or intramedullary nailing versus external fixation with conversion to internal fixation. A Lower Extremity Assessment Project (LEAP) found that type III open tibial shaft fractures that initially had higher rates of infections and nonunion were managed by external fixation compared to those initially managed by intramedullary nail fixation [1]. Another study, Modern Ring External Fixators Versus Internal Fixation, looked at patients with type III open tibia fractures who received either and internal or external fixation [1]. The results were like the LEAP study, showing no advantage of external fixation over internal fixation. There was a greater risk of loss of fracture reduction and device failure in the external fixation group [1]. Internal fixation, when possible, delivers more advantageous outcomes compared to external fixation.

Administering antibiotics as soon as possible after the injury can significantly reduce the risk of infection. This is because it can help eliminate bacteria before it can multiply and establish an infection as well as help prevent biofilm formation [22,23]. Early antibiotics reduce the risk of bacteria spreading from the site on injury to surrounding tissues or systemically [22]. Antibiotic administration as soon as possible is favorable and cost-effective. Delaying antibiotics for more than three hours after injury has been linked to a much higher risk of developing a secondary infection [1]. According to the American Academy of Orthopedic Surgery (AAOS), cefazolin or clindamycin are recommended as antibiotic prophylaxis [1,9]. For type III fractures, it is advised to add additional gram-negative coverage with an aminoglycoside. However, they have been associated with nephrotoxicity and ototoxicity [9]. There needs to be more evidence to determine their benefit.

Recent evidence challenges the historical belief that all open fractures require surgical within 6 hours, as the timing of debridement has not been shown to significantly impact infection rates or clinical outcomes [24]. Studies indicated that neither early debridement nor the timing of soft tissue coverage or bone grafting significantly affects rates of infection [1,25]. A systematic review and meta-analysis found no statistically significant association between delayed debridement and infection rate or nonunion rate [26]. On the other hand, the GOLIATH Meta-Analysis found the risk of infection in severe open fractures rises as the time to perform debridement increases [27]. A study in South India examined adults with severe open fractures to assess how the timing of debridement affected outcomes in a tropical climate. Patients who underwent debridement more than 12 hours after injury had a higher risk of infection and nonunion, with delays increasing infection risk [28]. This study highlights how the setting of a fracture influences treatment decisions, emphasizing the need to consider environmental factors when determining the best course of care [28].

While early surgery for high-risk contaminated fractures is preferred by many surgeons, there is debate in the current literature about whether treating open fractures should be considered an urgent orthopedic emergency to avoid the risk of infection [1].

Antibiotics are recommended preoperatively. The timing of debridement and wound closure is crucial according to the AAOS. Performing debridement within 24 hours by a skilled team is typically sufficient. Closing the wound during the initial surgery has been associated with lower infection and nonunion rates. When soft tissue reconstruction is required, it should ideally be completed within the first week. The AAOS strongly recommends irrigation with saline during initial wound management. Further, for open fractures, the AAOS recommend negative pressure wound therapy to decrease the possibility of a revision surgery or infection following a closed fracture fixation [29].

## Selection of Antibiotics following Open Fractures

Antibiotic selection plays a critical role in preventing infections following open fractures. Beta-Lactam Antibiotics such as penicillin and cephalosporins are commonly used against gram positive bacteria. Piperacillin-Tazobactam provides broad-spectrum coverage for gram-positive, gram-negative, and anaerobic bacteria. Clindamycin can be used for patients that are allergic to penicillin, and it is effective against gram-positive bacteria. Monobactams such as Aztreonam has activity against gram negative pathogens like Pseudomonas but no coverage for gram-positive or anaerobic [9].

Currently, the Eastern Association for Surgery of Trauma recommends covering gram-positive bacteria initially, then gram-negative coverage added for type III open fractures [9]. They also recommend adding penicillin for barnyard injuries. Vancomycin is used most for MRSA infections [9]. A retrospective analysis examined patients with open fractures treated over several years and found a 20% infection rate [30]. The most common infections included MRSA, gram-negative bacteria, and polymicrobial cases. Cephalosporins and beta-lactam agents were moderately effective against gram-positive bacteria, while gentamicin and vancomycin showed high sensitivity against gram-negative and gram-positive bacteria, respectively [30]. By providing appropriate coverage for common pathogens, antibiotics can improve outcomes and reduce complications. Continued research and adherence to evidence-based guidelines will help refine antibiotic strategies and enhance patient care in the treatment of open fractures.

The management of open fractures involves a comprehensive approach that includes early antibiotic administration, surgical debridement, wound care, and fracture stabilization. While some recent evidence has challenged the “6-hour rule” for debridement, early antibiotic intervention remains preferred for high-risk fractures. Internal fixation generally provides better outcomes compared to external fixation, which carries higher risks of complications. Other practices include saline irrigation and negative pressure wound therapy [8].

## Reason for Continued High Rates of Infections

**Figure 2:**
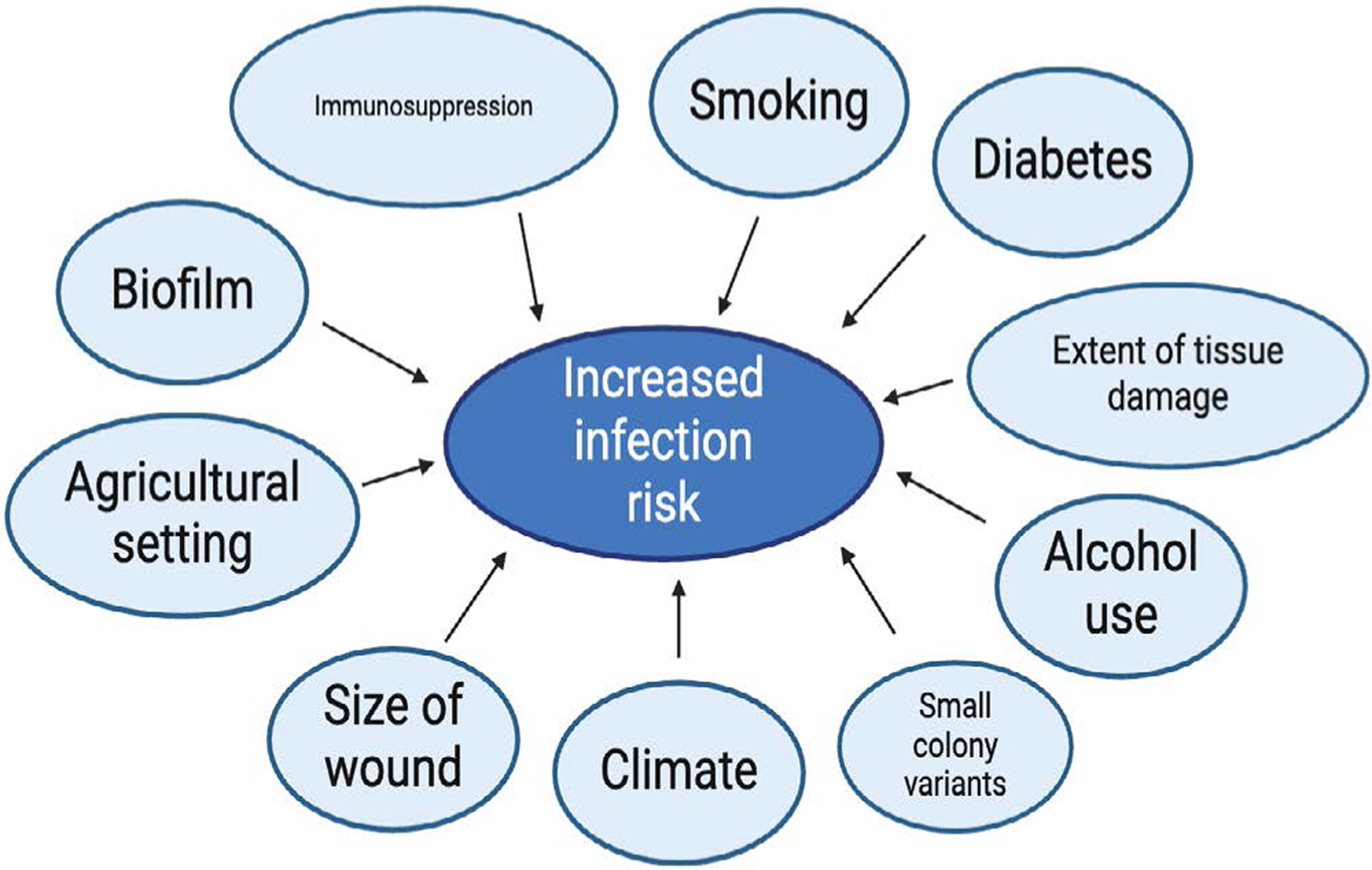
The figure shows various risk factors that can increase infection.

Despite best practices, open fractures still have a high incidence of infections. The rate can vary from 18 – 30% [31]. One common reason for the high incidence of infections is the formation of biofilms (Figure 2). These protect bacteria from host immune responses and antibiotics through the formation of structured communities of bacteria that adheres to surfaces like bone and implants [32]. They make it very difficult to treat infection and can lead to chronic, recurring infections. The structural integrity of biofilms is primarily due to its extracellular polymeric substances (EPS) matrix [32]. There is usually a high concentration of polysaccharides as well as proteins, nucleic acids, and lipids, within EPS that contributes to its stability. More characteristics of biofilms are its stickiness, which helps it withstand mechanical stress, and its viscoelasticity, which contributes to resistance of mechanical forces and aids in structural adaptation to allow for spreading [32]. It has been estimated that biofilm-associated infections account for 65–80% of human infections by the Center for Disease Control and National Institutes of Health [33]. Traditional antimicrobial therapies have shown limited success due to biofilms’ adaptive resistance mechanisms, which dynamically evolve to counteract specific pharmacological threats [34]. Biofilms can prevent antibiotics from interacting with bacterial targets, contributing to antibiotic resistance. The persistent challenge of biofilm in open fractures underscores the need for strategies that target their formation.

Another contributor to chronic infections and resistance is due to small colony variants (SCVs) (Figure 2). They are a slow-growing subpopulation of bacteria that form smaller than normal colonies. SCVs are less susceptible to antibiotics, like aminoglycosides, partly due to their low membrane potential. Further, they can reside within host cells, shielding them from antibiotics. Staphylococcus aureus SCVs are known to persist within host tissues and resist antibiotics. They can be difficult to detect with standard diagnostic methods due to their slow growth and atypical morphology. More research is needed to be able to understand and address their role in infections, to improve patient outcomes [32].

Further risks of infection are due to the complexity of the injury. Severe open fractures with extensive soft tissue damage and contamination are difficult to manage and have higher infection rates. Significant soft tissue damage compromises the local blood supply and impairs the immune response, which then lead to an environment beneficial to bacterial colonization and infection [32,35]. Many open fractures can occur in contaminated environments, such as agricultural settings, and are at high risk for infection due to the introduction of soil, water, or other organic material into the wound. This can increase the bacterial load and likelihood of infection [7,32]. These challenges highlight the importance of timely debridement, appropriate antibiotic coverage, and effective wound management in reducing the risk of infection in severe open fractures.

Other host factors can make managing infections challenging. Diabetes, smoking, and immunosuppression impair wound healing and increase infection risk [29]. The American Academy of Orthopedic Surgeons (AAOS) notes that both diabetes and smoking greatly increase the risk of infections in the surgical site [29]. Diabetic patients often struggle with slow wound healing and weakened immune defenses, while smoking reduces both tissue oxygen levels and immune function [29]. One study compared different risk factors in infected and uninfected groups following tibial plateau fractures. It showed that diabetes was significantly higher in the infected group at a rate of 68.18% compared the only 19.12% in the uninfected group (p < 0.001) [36]. Immunosuppressed individuals face an even greater risk because their ability to combat infections is compromised [36]. Obesity and alcohol use post operatively can both increase infection risk [29]. The same study as above showed a greater BMI in the infected cases (p = 0.006) [36]. There are many other factors that can increase the risk of an open fractures such as the presence of multi-drug-resistant organisms in the hospital setting, or a breach in sterility in the operating room, and even certain climates and geographical areas can have a higher prevalence of certain pathogens [29]. In conclusion, managing open fractures is complicated by various factors, which can increase a patient’s infection risk and impair wound healing (Figure 2). A multifaceted approach that takes these risk factors into account is important for minimizing infection rates and improving patient outcomes following open fractures.

## Treatment Methods of Infections in Open Fractures

To address the high rates of infections following open fractures, there are a variety of different approaches that can enhance infection control, improve patient outcomes, and reduce complications. These are discussed below.

### Bacteriophage Therapy

**Figure 3:**
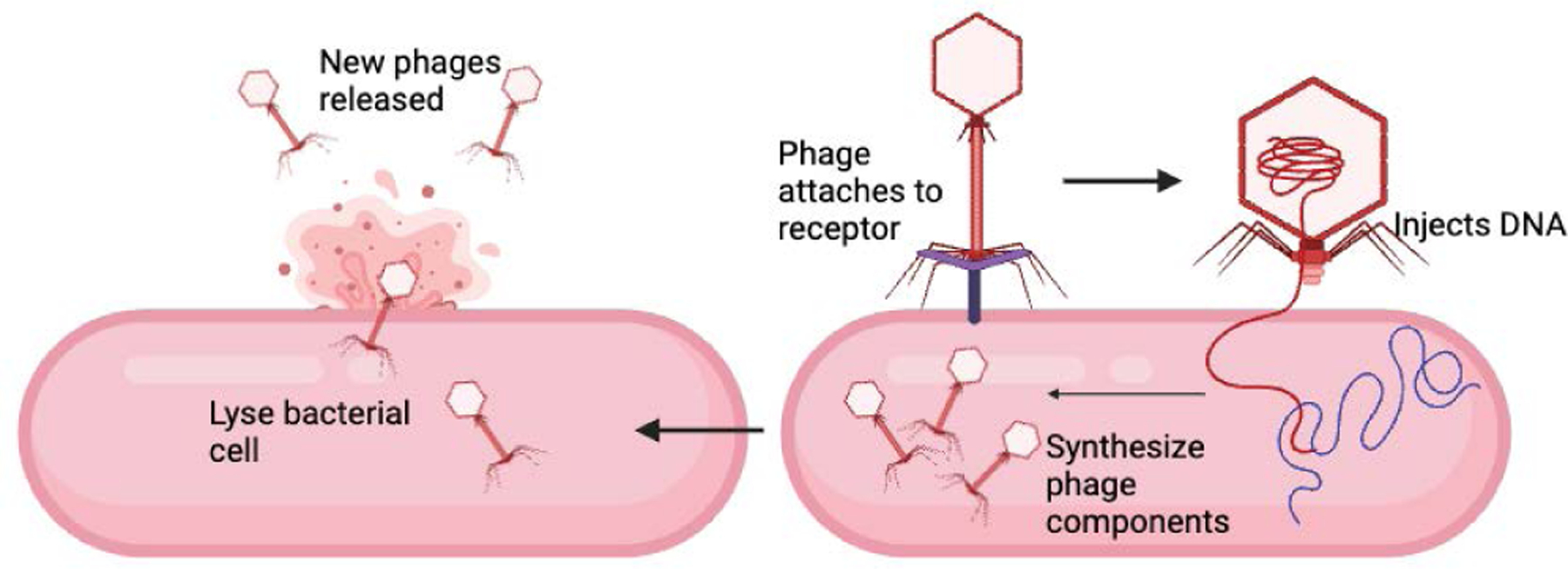
Process involved in phage-induced lysis of bacterial cell.

Bacteriophage Therapy is a promising alternative to traditional antibiotics and have been shown to have positive outcomes in treating complex fracture-related infections. While it was first introduced in 1917, there was a limited understanding of DNA and RNA as well as the introduction of antibiotics that negatively impacted the use of bacteriophages [37,38]. Currently, it is not approved by the US Food and Drug Administration (FDA), making it only available through the FDA’s expanded access program or clinical trials [39]. Bacteriophages, or phages, are viruses that infect, specifically target, and lyse bacterial cells [40]. Like all viruses, phages are dependent on a host to survive. While they contain all the information needed to replicate themselves, they lack machinery to produce energy or synthesize proteins. Therefore, they rely on infecting bacteria to carry out their metabolic functions [37]. There are several steps taken by phages that are necessary to lyse bacteria (Figure 3). First, they attach to the bacterial cells through receptors on the bacterial surface and the phage [41]. Next, they inject their DNA into the bacterial cells by degrading the peptidoglycan layer with phage lysosome activity and pore formation in the host cell wall. Once inside the cytoplasm, the phage DNA takes control of the host’s metabolic machinery and redirects the host’s resources towards replicating viral nucleic acid and producing phage proteins [41]. Newly synthesized viral components are assembled into complete phage particles. Finally, the bacterial cell is lysed through the actions of phage late proteins such as lysins, holins, or inhibitors of murein synthesis. This releases the new phages into the environment to infect other bacteria cells [41]. This repeats until the bacterial population is eliminated [40].

Phages can penetrate and disrupt bacterial biofilms, which are often resistant to antibiotics. Bacteriophages are highly selective, targeting single, specific bacterial strains while causing minimal disturbance to the overall microbial balance in the human body [42]. Most antimicrobial drugs have a broad spectrum and target a wide range of pathogens. This can have the potential negative effects of disrupting the normal microbial flora and may lead to secondary bacterial infections [42]. The precision of phages makes them a good alternative or complement to antibiotics with fewer side effects.

However, there are a few potential disadvantages to phage therapy. Unlike antibiotics, phage therapy requires precise identification of the bacterial species causing the infection by culturing a clinical sample and identifying the pathogen using standard microbiology diagnostics [41,43]. In addition, phages can be quickly eliminated by the host’s immune system, reducing their effectiveness in systemic infections [42].

Bacteriophage therapy shows promise in treating antibiotic-resistant bone and joint infections. Case studies and other research has highlighted its success in eradicating infections, promoting bone healing, and avoiding severe side effects. It has been effective in complex, multi-resistant infections and offers a targeted approach with minimal systemic reactions. Studies also demonstrate its ability to disrupt bacterial biofilms, a key factor in chronic infections. Standardization efforts are underway to optimize its use in musculoskeletal infections [41,42].

Bacteriophage therapy has demonstrated potential in treating complex bone and joint infections, including those caused by antibiotic-resistant bacteria. One case involved a patient with a severe infection following a gunshot injury who experienced successful bone healing without recurrence [43]. A broader review found that most patients achieved positive outcomes with minimal side effects [39,43]. Another study aimed at standardizing this therapy for musculoskeletal infections reported successful treatment without relapse or significant adverse reactions [44]. Bacteriophage therapy presents an encouraging alternative to traditional antibiotics, particularly in treating complex, antibiotic-resistant fracture-related infections. Studies have demonstrated its effectiveness in disrupting bacterial biofilms and providing targeted treatment, with high success rates and minimal systemic side effects.

There is limited clinical data on bacteriophage therapy, as there are majority case reports and small studies. Larger, well-controlled clinical trials are needed to establish efficacy and safety of bacteriophage therapy [45]. Many of the case reports and series showcase positive outcomes, with little to no documentation of instances where phage therapy was unsuccessful [40,43]. To optimize bacteriophage therapy in clinical use, this needs to be addressed through clinical trials.

### Antibiotic Coated Implants

The use of orthopedic implants in stabilizing open fractures increases the risk of infections. Antibiotic coated implants are orthopedic devices coated with antibiotics and used to prevent and treat infections. The antibiotics are released locally at the fracture site to maintain high local concentrations [46]. They break down bacterial cell membranes and biofilm structures, increasing the bacteria’s vulnerability to antimicrobial treatments and the body’s immune defense [47]. The coating can cause a sustained effect, depending on the coating material, it can last from days to weeks [47].

Gentamicin is typically used because studies have demonstrated that the amount released into circulation from the coated nails remains below levels that could cause systemic toxicity [46,48]. Studies also suggest that antibiotic-coated intramedullary nails help lower infection rates in open fractures, particularly in patients with comorbidities that increase their risk of infection [48,49]. While lowering the rate of infection, they can also help reduced days hospitalized and decrease additional operations, saving money [49].

Vancomycin and tigecycline can also be potentially used to coat implants [3]. One study investigated the use of antibiotic-coated intramedullary implants in open fractures to prevent *Staphylococcus aureus* infections. The results indicated that antibiotic coatings on implants can effectively reduce bacterial infection and the risk of osteomyelitis. Both vancomycin and tigecycline-coated implants were found to significantly decrease bacterial load, highlighting its potential in preventing infections in open fractures [3].

Silver coatings can also be used for the implants, in either high amount silver (MUTARS) and low amount silver (Agluna). Silver-coated implants are valuable due to their broad-spectrum antimicrobial properties, effectively targeting various bacterial strains [50–52]. They work by disrupting bacterial membranes, hindering DNA replication, and deactivating key enzymes through interactions with thiol groups. These coatings are generally biocompatible, posing minimal toxicity to surrounding tissues. Studies suggest that silver-coated implants are a safe option for medical use, as they do not lead to significant local or systemic adverse effects [50–52]. Another type are electrospun composite coatings which utilize nanofibers made from poly(ε-caprolactone) (PCL) and poly(lactic-co-glycolic acid) (PLGA), that are embedded with antibiotics like vancomycin and rifampicin [53]. This approach enables a controlled and prolonged release of antibiotics, improving their effectiveness against biofilm-related infections [53]. Finally, povidone-iodine coating can be applied to titanium plants, which provides antimicrobial properties and has shown good compatibility without the risk of developing resistance [54]. These implants can be coated using electrospinning methods, 3D printing and airbrush spray coating, hydrogel coatings, or a three-layer sandwich type coating [53,55–57].

Antibiotic coated implants represent an important strategy for reducing infection risk in open fractures. These coatings, whether antibiotic-based, silver-infused, or polymeric nanofiber structures, offer targeted antimicrobial effects while maintaining biocompatibility. Studies suggest that these coatings not only reduce bacterial colonization but also lower infection rates, shorten hospital stays, and minimize the need for additional surgeries. As research continues to refine these technologies, their widespread adoption could significantly enhance patient outcomes and improve open fracture related care.

### Bioelectric Dressings

As discussed earlier, a major complication from wounds that can arise is the development of biofilms which can form a protective matrix around individual microbes that make up the biofilm. As a result, alternative treatment strategies are needed to effectively combat biofilm-related infections.

Bioelectric dressings provide are a way to potentially treat open fracture infections that does not involve the use of antibiotics. They work by generating a low-level electric field that disturbs bacterial activity [58]. The dressing has electrodes that produce a continuous microcurrent. The mechanism typically involves the patterned deposition of silver (Ag) and zinc (Zn) on the dressing fabric. Typical technologies used are Wireless Electroceutical Dressing (WED) and Modular Adaptive Electrotherapy Delivery System (MAEDS) [58]. When moistened these elements generate a weak electric field without the need for an external power source. This electric field disrupts the biofilm matrix and bacterial cell membranes, leading to a reduction in biofilm integrity and bacterial viability and therefore making it hard for bacteria to grow, form biofilms, or develop resistance [34,59,60]. By breaking down these protective biofilms, bioelectric dressings improve the efficacy of antimicrobial treatments and facilitate wound healing. Additionally, these dressings accelerate wound closure by stimulating cellular processes such as proliferation, migration, and angiogenesis, mimicking the body’s natural healing mechanisms [61]. They also help modulate inflammatory responses, reducing chronic inflammation and creating a more favorable environment for tissue repair [62]. Furthermore, some bioelectric dressings possess intrinsic antibacterial properties by generating reactive oxygen species (ROS) or other antimicrobial agents, reducing the need for systemic antibiotics [60,63]. Together, these properties make bioelectric dressings a promising tool for improving wound healing and infection management. However, it should be noted that some patients may experience mild skin irritation, or discomfort at the site of application [60].

Research has shown that MAEDS and WED both in a porcine wound model can significantly accelerate wound healing and effectively break down biofilm clusters, restoring the protective barrier of the skin [34,58]. However, bioelectric dressings require specialized equipment and expertise. Like bacteriophage therapy, more clinical trials are needed to establish long-term efficacy and safety.

### Continuous Local Antibiotic Perfusion

A new technique, continuous local antibiotic perfusion (CLAP), sustains a consistent therapeutic antibiotic solution at the site for an extended duration, offering less invasiveness and fewer complications compared to other approaches [64]. Unlike traditional, local application methods, CLAP delivers a continuous flow of antibiotic solution directly near the contaminated area either by syringe pumps using dual lumen tubes or bone marrow needles that are placed as minimally invasively as possible, guided by negative pressure to target the site [64,65]. Within CLAP, there are two methods that can be applied together or separately: intra-soft tissue antibiotic perfusion (iSAP) and intramedullary antibiotic perfusion (iMAP) [65]. Gentamicin may be used as the continuously administered antibiotic [64,65,67]. Systemic administration of antibiotics may be necessary for areas that CLAP cannot reach sufficiently [64]. CLAP aims to minimize dead space by excising only biologically inactive tissue, apply negative pressure to all incisions and abscess sites to manage dead space, deliver a continuous infusion of appropriately concentrated antibacterial drugs directly to the infected area using negative pressure, and finally continuously drain antibacterial drugs through negative pressure to reduce their systemic side effects [64,65]. CLAP has been shown to effectively control infections in many cases, with no additional surgeries required in most [64,67].

Intramedullary antibiotic perfusion (iMAP) is used for bone infections, where a bone marrow needed is inserted to the suspected infectious area. Whenever possible, the needle is positioned to ensure the affected area is compressed or supported. A 2.4 mm Kirschner wire is used to create a hole in the cortical bone and then, using a hammer, a bone marrow needle is inserted. Inject contrast media to ensure the antibiotics reaches the infection site. One study iMAP was used in 10 cases to treat fracture-related infections after osteosynthesis. The results showed that in all patients, the developed infections were cleared, the patients’ implants retained, and there was fracture union without complications. These findings highlight the potential of iMAP as a reliable technique for treating fracture related infections [68].

Intra-soft tissue antibiotic perfusion (iSAP) is used for soft tissue infections and management of dead spaces. The tip of a dual-lumen tube releasing the antimicrobial should be positioned deep within the dead space to ensure the largest treatment area. The discharge holes of the tube should be distributed across a wide portion of the space. This setup allows the infected area to be thoroughly saturated with antibacterial drugs while applying consistent negative pressure to effectively drain the space and prevent dead space formation. A study employed iSAP in 10 patients with a favorable wound bed successfully prepared in all cases, with no recurrence of infection observed. These results demonstrate the effectiveness of iSAP in managing soft tissue infections and preventing complications, making it a valuable approach for open fracture related infections [69].

Continuous local antibiotic perfusion (CLAP) is a novel approach to local antibiotic administration, with iMAP and iSAP most beneficial when applied together. They allow for higher local concentrations of antibiotics at the site of infection while reducing systemic toxicity [64]. Some potential drawbacks are these methods require specialized equipment and expertise which may not be readily available in all healthcare settings. While there have been reports of potential side effects of CLAP, further research is needed to better understand and mitigate these risks, especially in patient with comorbidities or those at higher risk for complications [66,69]. Despite potential downsides, CLAP, iMAP, and iSAP offer significant advantages in managing open fracture infections.

## Conclusion

The management of open fractures presents significant challenges due to the high risk of infections, complications related to bacterial biofilms, and the increasing prevalence of antibiotic resistance. An understanding of open fracture classification systems, combined with a multidisciplinary approach to treatment, is essential for optimizing patient outcomes. Traditional strategies such as early surgical intervention, debridement, and antibiotic prophylaxis remain important in infection prevention. There are promising emerging therapies that address limitations of conventional treatments. Integrating new technologies with established surgical and antimicrobial protocols may pave the way for more effective and tailored approaches to managing complex open fractures. Research continues for each of these treatment options, and larger, well-controlled clinical trials are necessary to better understand their efficacy and safety in diverse patient populations. It would be highly informative to conduct clinical trials that look specifically at patients with high risk factors, such as diabetes or immunosuppression, who are more prone to infections following open fractures. These studies could provide insight into the effectiveness of novel treatments in mitigating infections rates and improving patient outcomes in these vulnerable groups. Further, it would be beneficial to develop tailored treatment protocols based on patient-specific factors, including comorbidities, and the severity of the open fracture. Personalized approaches could improve infection control and outcomes. In addition, research could investigate the potential of combining certain therapies to see if together, they provide more coverage for patients or synergistic effects that enhance patient outcomes. Another area of future study is to evaluate the long-term outcomes of these emerging therapies, with a focus on recurrence of infection, recovery and patient quality of life. Understanding these long-term implications could help optimize post-open fracture management. By focusing on these key areas, future research could contribute to more effective, individualized care for patients with open fractures and hopefully help reduce the burden of infection-related complications.

## Key Points

1. The Gustilo-Anderson Classification (GAC) system categorizes open fractures based on wound size, soft tissue damage, contamination, and vascular injury, with higher grade (Type III) having a significantly higher infection risk and worse prognosis.
2. The most common pathogens in open fractures include *Staphylococcus aureus*, *Staphylococcus epidermidis*, and gram-negative bacteria like *Enterobacter* and *Pseudomonas*, with a notable incidence of polymicrobial infections.
3. Open fractures have a consistent nonunion rate of 13–17% over four decades, with delayed healing in approximately 8% of cases, leading to prolonged disability, increased need for surgeries, and higher healthcare costs.
4. Immediate administration of antibiotics significantly reduces infection risk, and surgical debridement is essential, although recent studies challenge the historical 6-hour rule.
5. Internal fixation is generally favored over external fixation due to lower infection rates and better fracture stability.
6. Bacteria form protective biofilms that shield them from immune responses and antibiotics, leading to chronic infections and antibiotic resistance.
7. Bacteriophage Therapy selectively target and lyse bacteria, offering a promising alternative for treating antibiotic-resistant fracture-related infections.
8. Orthopedic implants coated with antibiotics release antimicrobial agents locally, reducing bacterial colonization and infection rates.
9. Bioelectric dressings generate microcurrents that disrupt bacterial biofilms, enhancing wound healing and antimicrobial effectiveness.
10. Continuous Local Antibiotic perfusion delivers antibiotics directly to infected areas using negative pressure, effectively treating infections while minimizing systemic side effects.
